# Acylcarnitine Profiles in Acetaminophen Toxicity in the Mouse: Comparison to Toxicity, Metabolism and Hepatocyte Regeneration

**DOI:** 10.3390/metabo3030606

**Published:** 2013-08-02

**Authors:** Sudeepa Bhattacharyya, Lisa Pence, Richard Beger, Shubhra Chaudhuri, Sandra McCullough, Ke Yan, Pippa Simpson, Leah Hennings, Jack Hinson, Laura James

**Affiliations:** 1 Departments of Pediatrics, University of Arkansas for Medical Sciences, Little Rock, AR 72202, USA; E-Mails: SBhattacharyya2@uams.edu (S.B.); chaudhurishubhra@uams.edu (S.C.); mcculloughsandras@uams.edu (S.M.); 2 Arkansas Children’s Hospital Research Institute, Little Rock, AR 72202 , USA; 3 Division of Systems Biology, National Center for Toxicological Research, Jefferson, AR 72079, USA; E-Mails: Lisa.Pence@fda.hhs.gov (L.P.); Richard.beger@fda.hhs.gov (R.B.); 4 Medical College of Wisconsin, Milwaukee, WI 53226, USA; E-Mails: kyan@mcw.edu (K.Y.); psimpson@mcw.edu (P.S.); 5 Departments of Pathology, University of Arkansas for Medical Sciences, Little Rock, AR 72205, USA; E-Mail: lhennings@uams.edu; 6 Departments of Pharmacology and Toxicology, University of Arkansas for Medical Sciences, Little Rock, AR 72205, USA; E-Mail: hinsonjacka@uams.edu

**Keywords:** acetaminophen, hepatic, β-oxidation, toxicity, acylcarnitine

## Abstract

High doses of acetaminophen (APAP) result in hepatotoxicity that involves metabolic activation of the parent compound, covalent binding of the reactive intermediate *N*-acetyl-p-benzoquinone imine (NAPQI) to liver proteins, and depletion of hepatic glutathione. Impaired fatty acid β-oxidation has been implicated in previous studies of APAP-induced hepatotoxicity. To better understand relationships between toxicity and fatty acid β-oxidation in the liver in APAP toxicity, metabolomic assays for long chain acylcarnitines were examined in relationship to established markers of liver toxicity, oxidative metabolism, and liver regeneration in a time course study in mice. Male B6C3F1 mice were treated with APAP (200 mg/kg IP) or saline and sacrificed at 1, 2, 4, 8, 24 or 48 h after APAP. At 1 h, hepatic glutathione was depleted and APAP protein adducts were markedly increased. Alanine aminotransferase (ALT) levels were elevated at 4 and 8 h, while proliferating cell nuclear antigen (PCNA) expression, indicative of hepatocyte regeneration, was apparent at 24 h and 48 h. Elevations of palmitoyl, oleoyl and myristoyl carnitine were apparent by 2–4 h, concurrent with the onset of Oil Red O staining in liver sections. By 8 h, acylcarnitine levels were below baseline levels and remained low at 24 and 48 h. A partial least squares (PLS) model suggested a direct association of acylcarnitine accumulation in serum to APAP protein adduct and hepatic glutathione levels in mice. Overall, the kinetics of serum acylcarnitines in APAP toxicity in mice followed a biphasic pattern involving early elevation after the metabolism phases of toxicity and later depletion of acylcarnitines.

## 1. Introduction

Acetaminophen (APAP) is the most widely used drug for the treatment of pain and fever around the world. While the drug is generally considered to be safe for use when administered in doses recommended by the manufacturer, at high doses, APAP produces fulminant hepatocellular necrosis and accounts for 50% of all cases of acute liver failure in adults and 14% of cases in children [1,2]. The role of metabolism in APAP toxicity is well characterized and involves generation of the highly reactive metabolite of APAP known as N-acetyl-p-benzoquinone imine (NAPQI) and hepatic glutathione (GSH) depletion [3]. NAPQI is formed through the oxidative metabolism of APAP and covalently binds to the amino acid cysteine in proteins, forming APAP protein adducts. APAP protein adducts are formed in the liver and other tissues during the early stages of APAP toxicity in mouse models [4,5] and released into peripheral blood upon hepatocyte lysis. Even though covalent binding and the formation of adducts is considered to be a prerequisite to the development of APAP toxicity, a large number of studies have indicated that other mechanisms are involved in the mediation of toxicity. Production of reactive oxygen/nitrogen species in hepatic mitochondria, mitochondrial permeability transition, and c-Jun N-terminal protein kinase activation are also recognized as mechanisms of APAP toxicity in the mouse model [6,7].

An evolving body of literature also supports the protective role of the peroxisome proliferator-activated receptor alpha (PPARα) in APAP toxicity [8,9,10]. This nuclear receptor is a member of the superfamily of nuclear receptors that controls the expression of genes encoding mitochondrial fatty acid β-oxidation. Pre-treatment of mice with PPARα ligands, such as clofibrate or WY-14683, was reported to produce resistance to APAP toxicity in mice. Chronic, two-week treatment with clofibrate [9,11] and single dose clofibrate treatment prior to APAP [12] attenuated toxicity in the mouse. However, two-week pre-treatment with clofibrate also inhibited covalent binding and increased APAP-glutathione levels in bile, showing that the PPARα agonist reduced sensitivity to APAP toxicity by altering the metabolism of APAP. In contrast, APAP metabolism was unaffected by pre-treatment with a single dose of clofibrate [12], suggesting the possible involvement of other mechanisms of hepatoprotection mediated through PPARα [13]. The mechanisms whereby PPARα activation modifies sensitivity to APAP are poorly understood. Cell proliferation is a known effect of PPARα activation and could potentially alter the sensitivity of mice to APAP, as suggested by two previous studies [10,13]. In addition to the effects of PPARα activation on lipid metabolism and gluconeogenesis, activation of this pathway is known to have anti-inflammatory effects by suppressing interleukin 1 and reducing NFκB activity [14].

Serum elevations of long-chain acylcarnitines, intermediates in the mitochondrial β-oxidation of fatty acids, have recently been used as an indicator of impaired fatty acid oxidation in liver in mouse models of APAP toxicity [8,15,16,17]. The β-oxidation of fatty acids is a well-known adaptive response to fasting or stress and represents a switch in the cellular energy source from glucose to free fatty acids [16,17]. Fatty acids are activated via cytosolic acyl co-A synthetase, and then transported into mitochondria via carnitine, concluding with β-oxidation within the mitochondrial matrix. Coen previously reported the elevation of triglycerides and monounsaturated fatty acids (MUFA) and the decrease in polyunsaturated fatty acids (PUFA) in liver from mice treated with a toxic dose of APAP using conventional solution state nuclear magnetic resonance spectroscopy [17]. These findings, accompanied by elevations of glucose, acetate, pyruvate, and lactate, suggested that APAP toxicity results in increased glycolysis, as well as a mitochondrial inability to use pyruvate in the citric acid cycle and impaired fatty acid β-oxidation in liver mitochondria [17]. In addition, Chen and Gonzalez previously suggested that suppression of PPARα occurs in APAP toxicity and noted early increases in serum palmitoyl carnitine, that was attributed to a disruption in fatty acid β-oxidation in liver mitochondria [16]. Palmitoyl carnitine levels were increased in wild type mice treated with APAP and were reduced, but not absent, in CYP2E1 null mice, while hepatic GSH depletion, reflecting the drug metabolism, was comparable in the two groups of mice. CYP2E1 is the primary CYP450 involved in the metabolism of APAP. The relatively decreased (but not absent) serum levels of palmitoyl carnitine in the CYP2E1 null mice implicate the potential involvement of other CYP P450’s such as CYP3A4 or CYP1A2 in the oxidative metabolism of APAP. In addition, previous studies showing that the administration of carnitine, the carrier for fatty acids into mitochondria, provided protection in APAP toxicity, suggest an interaction between fatty acid oxidation and the development of toxicity [18,19].

In the mouse model and in clinical samples, APAP protein adducts in liver or serum has been shown to be a very sensitive and specific biomarker of APAP-induced metabolism and hepatotoxicity [20,21,22]. To further examine the relationship of metabolism (as reflected by depleted hepatic GSH and formation of APAP protein adducts) to markers of fatty acid oxidation in APAP toxicity in the mouse, long-chain acylcarnitines were measured using a targeted metabolomics approach and examined in relationship to the known indicators of toxicity, metabolism, and liver repair. The data presented herein illustrate the time course of disruptions of fatty acid β-oxidation in liver and subsequent changes in serum acylcarnitines in relationship to early and late phase indicators of APAP toxicity, metabolism, and recovery.

## 2. Results and Discussion

### 2.1. Indicators of Hepatotoxicity and Oxidative Metabolism in APAP Treated Mice

**Figure 1:**
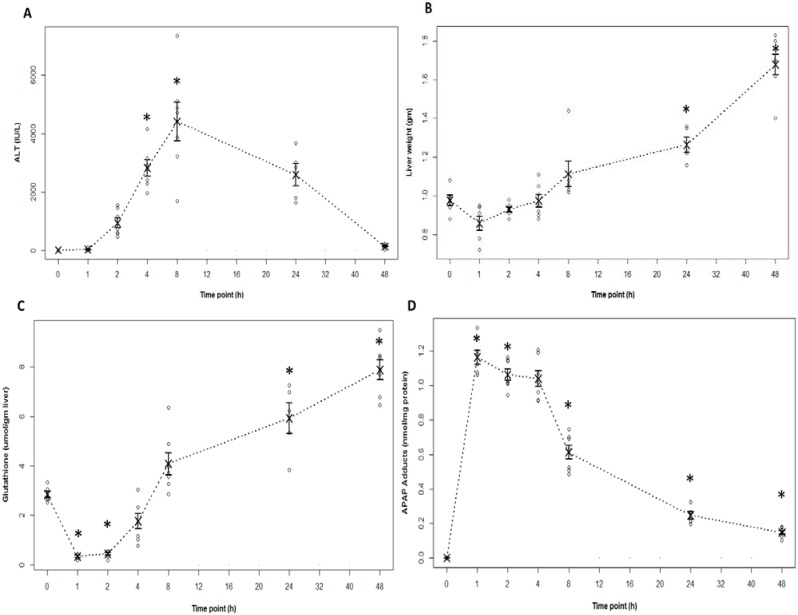
Indicators of toxicity and metabolism in B6C3F1 male mice treated with acetaminophen (APAP) (200 mg/kg IP). (**A**) Serum alanine aminotransferase (ALT) was increased above control at 4 and 8 h (* represents statistical significance at *p* < 0.05 in comparison to control mice, time 0). (**B**) Liver weights were increased at 24 and 48 h. (**C**) Hepatic glutathione (GSH) was depleted by 1 h and remained low at 2 h (*p < 0.05) compared to control mice and returned to baseline values by 8 h. (**D**) Liver APAP protein adducts were increased by 1 h (* *p* < 0.05) and remained elevated at all time points compared to saline control mice). Raw data points are represented as “○” in strip plots; mean values represented as “×”, and standard error of the mean represented as capped bars.

**Figure 2:**
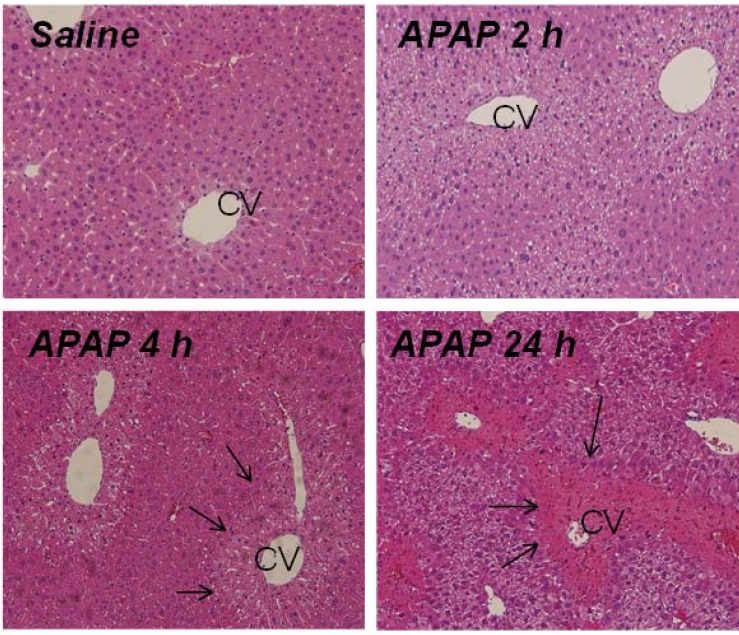
H and E stained liver sections of representative B6C3F1 male mice treated with APAP (200 mg/kg IP). By 2 h, centrilobular pallor was apparent in the cells surrounding the central vein (CV). By 4 h, frank necrosis (demonstrated by arrows) was present in the region surrounding the CV, which extended to bridging necrosis at 24 h post-APAP.

Following an overnight fast, B6C3F1 male mice were treated with APAP 200 mg/kg IP and sacrificed at 1, 2, 4, 8, 24 or 48 h. This dose of APAP produces significant toxicity without lethality and allows for study of both early (metabolism and liver injury) and late events (hepatic regeneration) in toxicity. [23] Control mice were also fasted overnight and treated with saline by IP injection. Consistent with previous studies [5,6,23,24] measurement of serum ALT, the biochemical indicator of liver injury, indicated significant elevation by 4 h and peak elevation at 8 h following the administration of APAP (Figure 1A). Histologic review of mouse liver sections indicated centrilobular pallor in hepatocytes at 2 h, followed by frank hepatocellular necrosis at 4 h, consistent with the ALT data (Figure 2). The extent of the hepatocellular necrosis progressed to the pattern of bridging necrosis and frank hemorrhage by the 24 h time point. Measurement of liver weight is a surrogate marker of liver injury and increases throughout the time course of toxicity due to hemorrhage and the pooling of blood in liver sinusoids. As shown in Figure 1B, liver weight was significantly increased at 24 and 48 h.

Measurement of hepatic GSH and APAP protein adducts reflect the role of oxidative metabolism in APAP toxicity. Consistent with previous data [24] measurement of hepatic GSH in the mice indicated marked depletion of hepatic GSH by 90% at 1 and 85% at 2 h compared to control (0 h) mice (Figure 1C). In addition, HPLC-EC analysis of APAP protein adducts in the supernatants of liver homogenates indicated a 10-fold elevation in adducts at 1 h, followed by a very gradual decline over the time course of the study (Figure 1D). Adduct levels at 48 h remained above baseline (Figure 1D). These data demonstrate that hepatic GSH depletion and the formation of APAP protein adducts are early events in APAP toxicity.

### 2.2. Indicators of Hepatocyte Regeneration in APAP Toxicity

**Figure 3:**
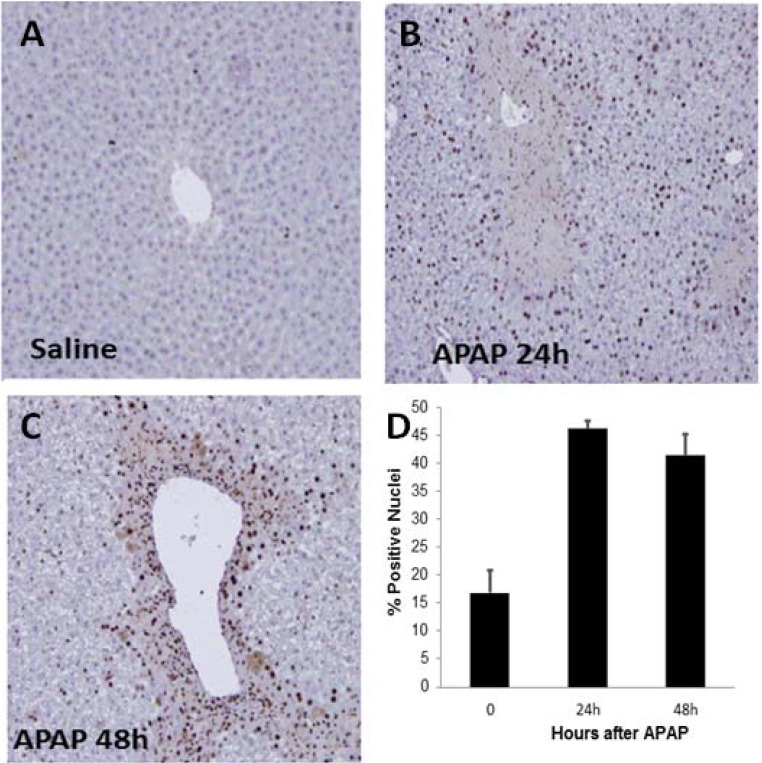
Immunohistochemical staining for proliferating cell nuclear antigen (PCNA) in B6C3F1 male mice treated with APAP (200 mg/kg IP). (**A**) No staining for PCNA was observed in saline treated mice. (**B**) PCNA staining (blown nuclear stain) was increased in the nuclei of hepatocytes throughout the midzonal and centrilobular regions of the liver at 24 and 48 h (**C**). (**D**) Summary data reflecting the percent increase in positive hepatocyte nuclei at 24 and 48 h compared to control (0 h).

Proliferating cell nuclear antigen (PCNA) expression is an established marker of cells undergoing proliferation that indicates cellular replication, expressly the G1 and S phases of mitosis. It is a commonly used indicator of hepatocyte regeneration in APAP toxicity [23]. In previous work, immunostaining for PCNA of hepatic liver sections demonstrated the initial appearance of proliferating hepatocytes at 24 h after APAP in the midzonal regions of the liver, bordering the regions of hepatocellular necrosis [23]. In the present study quantitative image analysis of PCNA stained hepatic nuclei indicated significantly increased staining at 24 h, which progressed in extent through the midzonal regions of the liver at 48 h compared to minimal staining in the control mice (Figure 3), consistent with previous data [25].

### 2.3. Oil Red O (ORO) Analysis and Liver Triglycerides

**Figure 4:**
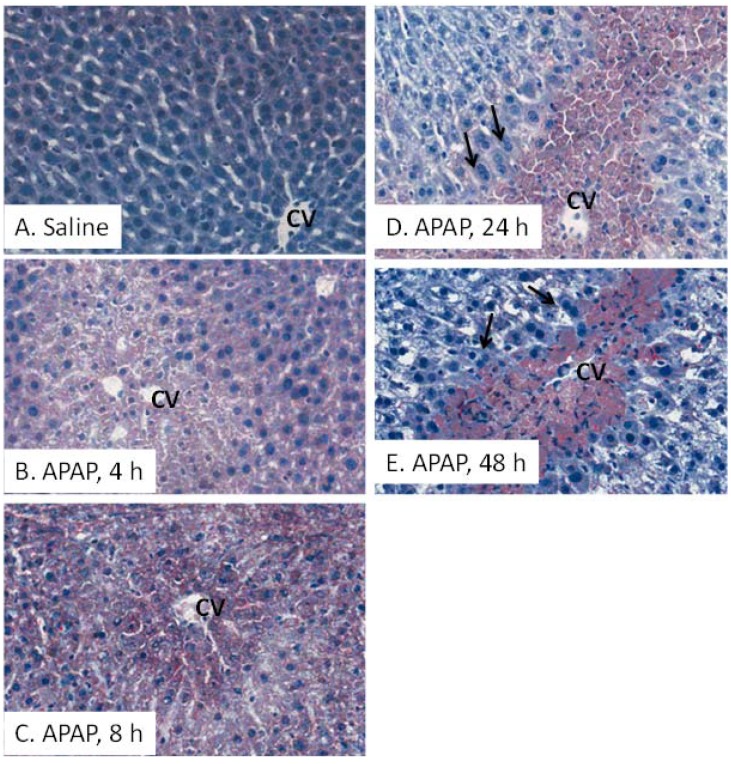
Oil Red O (ORO) staining in saline and APAP treated mice. Representative slides of ORO staining of mice treated with saline (**A**) or 200 mg/kg APAP and sacrificed at 4 (**B**), 8 (**C**), 24 (**D**) or 48 h (**E**). A diffuse pattern of ORO staining, indicative of hepatic lipid accumulation, was observed at 2 h (not shown) and peaked at 4 h. By 24 h, ORO staining was localized to the necrotic regions, adjacent to areas of regenerating hepatocytes (arrows).

Figure 4 illustrates ORO immunohistochemical images of mouse liver. Minimal staining for ORO was detected in the liver sections of the mice treated with saline (**A**). In contrast, ORO staining was diffusely present throughout the hepatic lobule by 2 h (not shown) and peaked at 4 h (B). At 8 h, the staining decreased in extent (C). By 24 and 48 h, the staining was localized to the regions surrounding the hepatic vein (D, E), bordering the areas of ongoing hepatocyte regeneration (Figure 4, arrows). The temporal sequence and pattern of ORO staining with the localization of ORO to the centrilobular regions suggested that lipid deposition also occurred in the later stages of toxicity and coincided with the time periods of hepatocyte regeneration (Figure 3) above.Elevations of hepatic triglycerides have been reported in APAP toxicity and likely reflect a switch in energy metabolism that occurs during the toxicity [16,26]. Hepatic triglyceride levels were not elevated in the APAP treated mice in the present study, likely as a result of the lower dose of APAP utilized (data not shown). 

### 2.4. Acylcarnitine Profiles in APAP Toxicity in the Mouse

**Figure 5:**
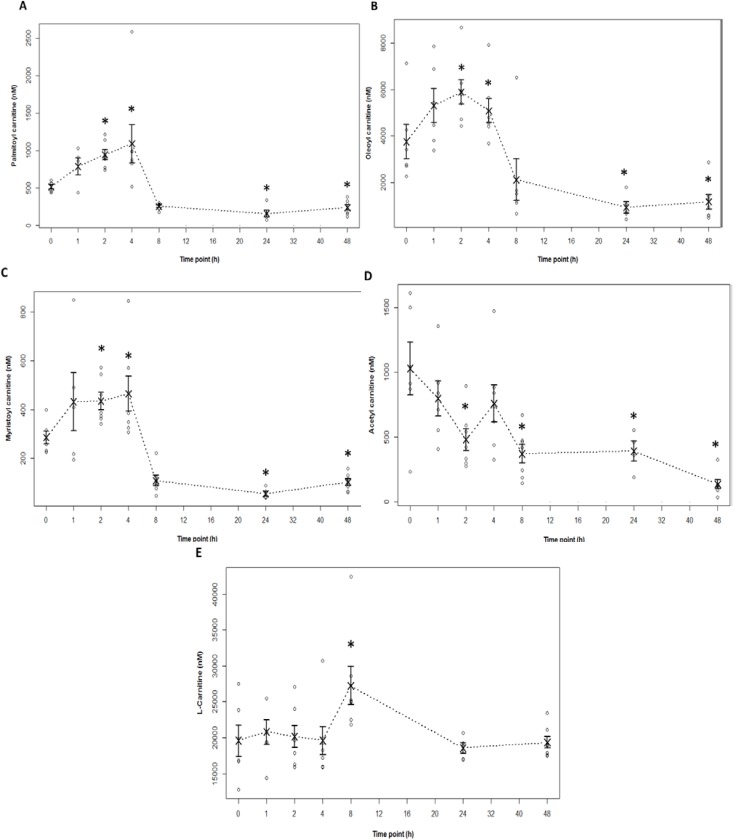
Serum acylcarnitine levels in mice treated with saline or APAP 200 mg/kg. The long chain acylcarnitines, palmitoyl (**A**), oleoyl (**B**) and myristoyl carnitine (**C**) showed statistically significant increases at 2–4 h (*indicates statistically significant at *p* < 0.05) compared to saline control (time 0 h), and decreased below baseline levels at 8–24 h before approaching baseline at 48 h. Acetyl-L-carnitine (**D**) significantly decreased over time compared to saline control, and L-carnitine (**E**) was elevated only at 8 h. Raw data points represented as “○”in strip plots; means as ““×”and standard error of the mean as capped bars at each time point.

Targeted metabolomic analysis was performed to determine changes in serum acylcarnitine profiles following dosing of mice with APAP 200 mg/kg. A biphasic pattern for long chain acylcarnitines was observed following treatment of mice with APAP. Among the long chain acylcarnitines, palmitoyl, oleoyl and myristoyl carnitines showed statistically significant increases by 2 h, and peaked by 4 h (Figure 5A–C). At 8 h and at later times, the levels fell significantly below the values observed in the control (0 h) mice. L-Carnitine levels were increased at 8 h compared to saline controls (0 h), while acetyl-L-carnitine showed a downward trend across all time points compared to saline controls (0 h) (Figure 5D,E). 

Overall, the acylcarnitine elevations were gradual and modest (doubling of approximately two fold) and appeared to follow the observed changes in markers of metabolism, as reflected by hepatic GSH and APAP protein adducts (Figure 1C,D).

Using a quantitative approach, [16] Chen and Gonzales observed similar elevations in palmitoyl carnitine in 129/Sv mice treated with 400 mg/kg of APAP. However, the biphasic pattern noted in the present study (Figure 5) was not observed by Chen *et al.* [16] Chen found that serum palmitoyl carnitine levels peaked at 2 h to a median value of approximately 580 nM, and remained above baseline at 24 h. A similar, but more modest elevation of palmitoyl carnitine was observed in CYP2E1 null mice and levels returned to baseline by 8 h, but did not fall below baseline at 24 h [16]. Differences in the temporal patterns of palmitoyl carnitine in the present study compared to the data of Chen *et al.* [16] may be multi-factorial in nature and could be secondary to differences in APAP dose (200 mg/kg *versus* 400 mg/kg), age (6 weeks *versus* 2 to 3 months), diet or the length of fasting time prior to the performance of the experiments, mouse strain, or differences in analytical approaches. Fasting is known to affect β-oxidation and thus, would affect acylcarnitine profiles [17]. In the present study, both the APAP treated and saline control mice were fasted for a 16 hour period of time before receiving APAP. In contrast, the mice used in Chen’s study were fasted for 24 h, while the saline mice were not fasted. Based on the time course data (Figure 5), oleoyl- and myristoyl-carnitine were comparable to palmitoyl-carnitine as biomarkers of disrupted fatty acid oxidation in APAP toxicity, and thus, complement and extend previous observations in this area [16].

**Figure 6:**
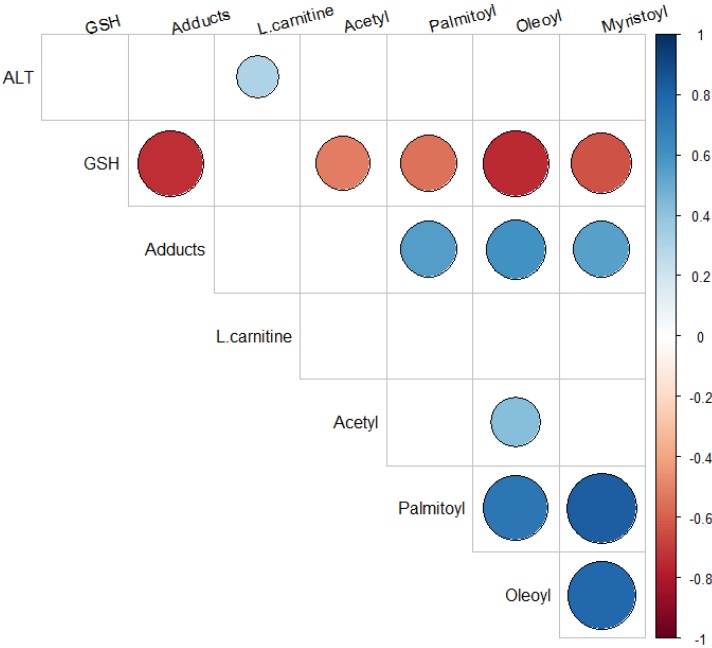
Spearman rank correlation heatmap between APAP toxicity and metabolism parameters and the acylcarnitines. Statistically significant correlations (p value < 0.05) are shown in blue (positive correlation) and red (negative correlation) color scale. Insignificant correlations are left blank. The larger the circle is the stronger is the correlation.

Spearman rank correlation analysis showed strong correlation (correlation coefficient > 0.81 at *p* values < 2E-10) among the long-chain acylcarnitines (Figure 6,) as would be expected based on the similarity of their structure and common routes of metabolism. In addition, our data showing below normal levels of acylcarnitines at later time points in the toxicity may have reflected heightened fatty acid oxidation to meet ongoing energy demands during the repair of liver injury and onset of hepatocyte regeneration [27]. In support of this postulation, previous data have reported increases in mRNA transcripts for carnitine palmitoyl transferases I and II occur in regenerating livers and that the administration of carnitine accelerates liver repair [27]. In addition, the upregulation of “carnitine shuttle” genes (e.g., carnitine-acylcarnitine translocase, carnitine palmitoyltransferase) has been reported in rats treated with triiodothyronine, a hepatocyte mitogen [28]. Coen used ^1^H NMR and showed changes in proton resonance peaks related to MUFA in the lipid extracts of liver tissue of mice treated with 500 mg/kg APAP [17]. Dose-response changes were demonstrated as well, and through PCA analysis, doses of 150 and 500 mg/kg APAP clearly separated from control and the 50 mg/kg APAP group. Our data demonstrated that a modest dose of APAP (200 mg/kg) is sufficient to impair fatty acid oxidation.

### 2.5. Relationship of Acylcarnitines to APAP Protein Adducts and Hepatic Glutathione

**Figure 7:**
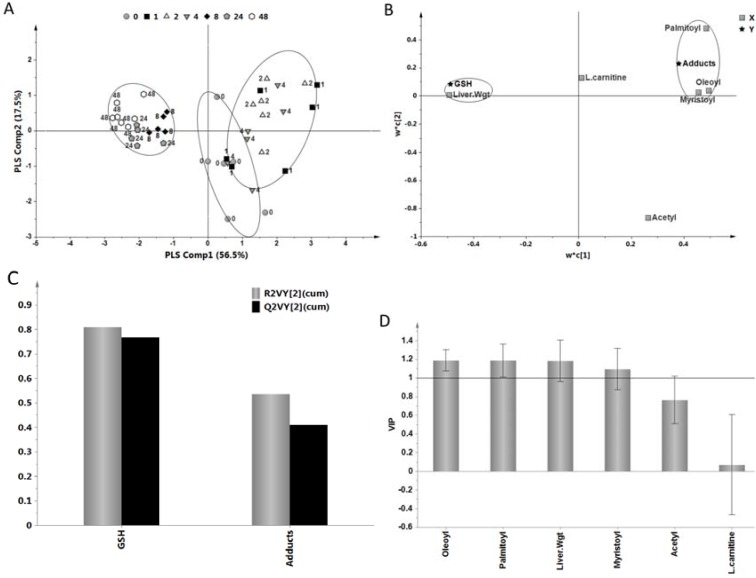
Partial least squares regression model describing the relationship between l-carnitine, acetyl, palmitoyl, oleoyl, myristoyl carnitine, liver weight, hepatic GSH and APAP protein adducts. (**A**) Scores plot of the samples showing the separation between the different time point groups (6 animals per group) along the two PLS components. The 8, 24 and 48 h time points clearly separated from the earlier time points (1, 2 and 4 h) and pre-dose (0) along the first PLS component. The goodness of fit (R^2^) and goodness of predictability (Q^2^) of the model were ~74% and 58.9%, respectively. (**B**) The corresponding loadings plot of the model showing the relationship between the acylcarnitine predictors and the toxicity/metabolism markers along the two partial least squares (PLS) components. The plot shows that palmitoyl, oleoyl and myristoyl carnitine correlated well to APAP protein adducts, while L-carnitine had minimal contribution to the model. (**C**) Explained variation of the two responses after the two PLS components in the model. Hepatic GSH and APAP protein adducts were well explained by the model and GSH performed slightly better based on the R^2^V(Y) and Q^2^V(Y) values. (**D**) VIP plot of the predictors showing their pooled contribution over the PLS component and all three responses. Palmitoyl, oleoyl, myristoyl carnitine, and liver weight had significant VIP values ( >1).

A multivariate, partial least squares regression (PLSR) model was used to examine relationships between acylcarnitines and APAP metabolism and toxicity parameters. This model captures the maximum covariance that exists in the data in the X (predictor variables) and Y (response variables) space. The two component PLS model (Figure 7A) captured 74% of the variation in the X variables (acylcarnitines and liver weight) that correlated with ~67.2% of the variation in the response variables (adducts and GSH). The goodness-of-predictability parameter (Q^2^(Y)) was 58.9%, which was calculated by a seven-round internal cross-validation of the data. The model was further validated by repeated permutations of the sample identifiers. A PLS scores plot (Figure 7A) demonstrated a clear separation along the first PLS components between the pre-dose, 0, early time points, 1–4 h, during which the changes in APAP toxicity parameters and acylcarnitines were maximal and the later time point groups, 8–48 h, during which all acylcarnitines were below baseline. The corresponding loadings plot, which summarizes the influence and correlation structure between the variables in the X and Y data, is shown in Figure 7B. X variables with large weights, positive or negative, were far away from the origin and highly correlated with Y, while Y variables with large weights were highly correlated with X. As shown in the loading plot, palmitoyl-, oleoyl-, myristoyl-carnitine, and liver weight were significant contributors to the separation in the earlier and later time point groups, while L-carnitine, positioned close to the origin, had minimal contribution to the model. Moreover, palmitoyl, oleoyl and myristoyl closely correlated with adduct values, while liver weight (Figure 1B) correlated with hepatic GSH, reflecting primarily the association of rebounding GSH (Figure 1C) in the later stages of APAP toxicity. The Variable Importance on the Projection (VIP) parameter summarizes the importance of the predictor variables both to explain X and to correlate to Y. Palmitoyl, oleoyl, and myristoyl carnitine, as well as liver weight, had VIP values above 1, indicating significant contribution to the model (Figure 7D). An overview of the explained variation of the two response variables by the two-component PLS regression model is presented in Figure 7C indicating slightly better modeling of GSH in comparison to adducts, based on the higher R^2^VY and Q^2^VY values in the former. As would be expected, the inclusion of ALT as a response variable in the PLS model resulted in dramatic decrease in the R^2^ and Q^2^ values (data not shown), indicating a poor association of ALT with the acylcarnitines in APAP induced toxicity. ALT is known to be a relatively “late stage” indicator of toxicity. Overall, the PLS model suggested a direct association of palmitoyl, oleoyl and myristoyl carnitine accumulation in serum to changes in APAP protein adduct levels and hepatic GSH levels in mice treated with APAP.

## 3. Materials and Methods

### 3.1. Drugs and Reagents

APAP was obtained from Sigma-Aldrich (St. Louis, MO, USA). Optima LC/MS grade water, acetonitrile formic acid, carnitines and all MS standards were purchased from Fisher Scientific (Hampton, NH, USA).

### 3.2. Experimental Animals

Six-week old male B6C3F1 mice (mean weight, 25.1 g) were obtained from Harlan Sprague Dawley (Indianapolis, IN). All animal experimentation was in accordance with the criteria of the “Guide for the Care and Use of Laboratory Animals” prepared by the National Academy of Sciences. Protocols for animal experimentation were approved by the University of Arkansas for Medical Sciences Animal Care and Use Committee. Mice were acclimatized one week prior to the planned experiments and fed *ad libitum*. Animals were housed 3 per cage and maintained on a 12 h light/dark cycle. On the day prior to experiments, mice were fasted overnight and dosing studies began at 0800 the following morning. Food was returned to the mice 4 h after either APAP or saline treatment. APAP was administered at 200 mg/kg IP and control mice received saline IP, designated as time 0. Mice were sacrificed to harvest liver tissue at 1, 2, 4, 8, 24 or 48 h (6 mice/group) after APAP. Animals were anesthetized with CO_2_ for blood sampling. Blood was removed from the retro-orbital plexus, allowed to coagulate at room temperature, centrifuged, and the serum was aliquoted for measurement of alanine aminotransferase (ALT) and metabolomic analysis of serum acylcarnitines. Mice were then euthanized in a CO_2_ atmosphere followed by cervical dislocation and removal of the livers. The livers were weighed and a portion was preserved in formalin for histological sections. The remaining livers were snap frozen in liquid nitrogen and stored at −80 °C for additional analyses.

### 3.3. Assessment of Hepatotoxicity

Serum ALT levels, a widely used indicator of liver injury, were measured using an Alera chemistry analyzer (Alfa Wassermann, West Caldwell, New Jersey).

### 3.4. Determination of Hepatic Glutathione and APAP Protein Adducts

Quantitation of hepatic glutathione (GSH) was performed using Ellman’s reagent following standard protocols [29]. APAP covalently bound to protein in liver was measured by initial protease treatment of liver homogenates followed by high performance liquid chromatography-electrochemical (HPLC-EC) analysis for APAP cysteine as previously described [22,30] 

### 3.5. Oil Red O Analysis of Hepatic Lipid Deposition

Lipid deposition in mouse liver was assessed by Oil Red O (ORO) staining of frozen hepatic sections [31]. For quantification of ORO, liver sections were scanned to a high-resolution digital file (Aperio Scanscope CS system; Aperio, Vista, CA) at 200× total magnification. A positive-pixel count algorithm within the Aperio system was optimized and used to express ORO counts (red pixels) relative to the total slide image. To better visualize positive pixels, a digital mask was applied to identify negative (blue), weakly positive (yellow), moderately positive (orange), and strongly positive (red) pixels. 

### 3.6. Proliferating Cell Nuclear Antigen (PCNA) Immunohistochemistry

Immunohistochemical assays for PCNA in liver sections was performed as per our previous publication [23], using a mouse monoclonal PCNA antibody (Dako, Carpinteria, CA) (1:75) and Gills Hematoxylin II as the counterstain. Quantification of hepatocyte proliferation (PCNA) was performed using the Aperio Scanscope described above. Algorithm settings were optimized for PCNA liver staining. PCNA-labeled hepatocytes were analyzed over the entire slide using the nuclear algorithm and color deconvolution to analyze chromagen staining. Hepatocyte nuclei were recognized by shape, size, and staining characteristics. Results were reported as percent positive nuclei. The pathologist was blinded to the treatment times of the groups.

### 3.7. Targeted Analysis of Serum Acylcarnitines Using a UPLC-TQ

Samples were removed from storage at −80 °C and placed at −4 °C for two hours. 50 μL of serum was spiked with isotope labeled L-carnitine, acetyl-L-carnitine, myristoyl-carnitine, palmitoyl carnitine and oleoyl carnitine. The sample was then de-proteinized with 300 μL of 3:1 mixture of Acetone:MeOH (Optima grade, Fisher scientific) and centrifuged for 15 minutes at 13,000 × *g* (4 °C). The supernatant was transferred into a total recovery auto-sampler vial (Waters, Milford MA) and the solvent was evaporated under a stream of nitrogen. Samples were reconstituted in 50 μL of 50:50 acetonitrile/75% methanol. The chromatographic separation was done using an Acquity UPLC equipped with a BEH C8 column (2.1 × 100 mm, 1.7 μm particle size) and VanGuard C8 pre-column (Waters). 10 μL was injected and the flow rate was set to 0.4 mL/min with a column temperature of 40 °C. Mobile phase A consisted of 90:10 water:acetonitrile with 0.5 g/L ammonium acetate adjusted to pH 7.4. Mobile phase B consisted of acetonitrile with 0.5 g/L ammonium acetate. The gradient started with 50% mobile phase B, held for 1 minute, increasing to 86% at 5 minutes then to 100% at 7 minutes, after which initial conditions were restored at 8.5 minutes and the system was equilibrated for an additional 1.5 minutes. Blank injections were made between each sample injection. Mass spectrometric analysis was done on a Xevo triple-quadrupole instrument (Waters) operated in positive electrospray ionization mode. Multiple Reaction Monitoring (MRM) was carried out using transitions previously optimized for each carnitine species by the direct infusion of standards. The capillary voltage was 4.5, the source and desolvation temperature were 150 °C and 400 °C, respectively. The desolvation gas flow rate was 800 L/h.

### 3.8. Data Analysis

Non-parametric Mann Whitney U-Test was used to detect differences between each time point group *vs.* baselines for all toxicity and metabolic markers using SPSS Version 10.0 (SPSS Inc, Chicago, IL) and the open source statistical software package R. A *p* value < 0.05 was considered significant for all analyses. Spearman rank and/or Pearson’s correlation was calculated between the measures of average intensity and positivity from ORO staining and acylcarnitines. Correlation analysis was also performed to examine the relationship between the metabolites and the recognized markers of APAP toxicity and metabolism. Multivariate data analysis was performed by the R package chemometrics [32] and SIMCA-P+ software (Umetrics, Kinnelon, NJ, USA). The data matrix was transformed by autoscaling and a partial least squares (PLS) regression model was generated in order to identify the acylcarnitines that closely associated with the toxicity markers. Major latent variables in the data were represented in a scores scatter-plot and the significant predictors were identified by their contribution to the PLS vectors in the loadings scatter plot as well as based on their variable importance on the projections (VIP) scores. Data are presented as mean ± standard error of the mean. 

## 4. Concluding Remarks

The present study was conducted to examine the temporal relationships between known indicators of APAP toxicity and metabolism (hepatic GSH and APAP protein adducts) and recognized intermediates of fatty acid β-oxidation in mitochondria, the long-chain acylcarnitines. High-resolution LC-MS based targeted metabolomics identified several serum long chain acylcarnitines (palmitoyl, myristoyl and oleoyl carnitine) which were elevated early in the toxicity following early changes in APAP adduct levels and hepatic GSH. APAP protein adducts in liver remained elevated through the time course of the toxicity. In the later stages of toxicity, at the time of liver recovery and hepatocyte regeneration, acylcarnitines fell below base-line, possibly due to heightened energy needs associated with the hepatocyte regeneration stages of toxicity. The data have relevance for future studies of APAP toxicity and support the role of impaired fatty acid β-oxidation as one mechanism in the development of toxicity. Understanding the relationship of drug metabolism to impaired β-oxidation is relevant to future studies focused on identifying human susceptibility to APAP toxicity, in particular for pathologic conditions associated with alterations in metabolic homeostasis and energy supply.

## References

[1] Larson A.M., Polson J., Fontana R.J., Davern T.J., Lalani E., Hynan L.S., Reisch J.S., Schiodt F.V., Ostapowicz G., Shakil A.O. (2005). Acetaminophen-induced acute liver failure: Results of a United States multicenter, prospective study. Hepatology.

[2] Squires R.H., Shneider B.L., Bucuvalas J., Alonso E., Sokol R.J., Narkewicz M.R., Dhawan A., Rosenthal P., Rodriguez-Baez N., Murray K.F. (2006). Acute liver failure in children: the first 348 patients in the pediatric acute liver failure study group. J. Pediatr..

[3] Mitchell J.R., Jollow D.J., Potter W.Z., Davis D.C., Gillette J.R., Brodie B.B. (1973). Acetaminophen-induced hepatic necrosis. I. Role of drug metabolism. J. Pharmacol. Exp. Ther..

[4] Cohen S.D., Khairallah E.A. (1997). Selective protein arylation and acetaminophen-induced hepatotoxicity. Drug Metab. Rev..

[5] Roberts D.W., Bucci T.J., Benson R.W., Warbritton A.R., McRae T.A., Pumford N.R., Hinson J.A. (1991). Immunohistochemical localization and quantification of the 3-(cystein-S-yl)-acetaminophen protein adduct in acetaminophen hepatotoxicity. Am. J. Pathol..

[6] Hinson J.A., Pike S.L., Pumford N.R., Mayeux P.R. (1998). Nitrotyrosine-protein adducts in hepatic centrilobular areas following toxic doses of acetaminophen in mice. Chem. Res. Toxicol..

[7] Jaeschke H., Gores G.J., Cederbaum A.I., Hinson J.A., Pessayre D., Lemasters J.J. (2002). Mechanisms of hepatotoxicity. Toxicol. Sci..

[8] Chen C., Hennig G.E., Whiteley H.E., Corton J.C., Manautou J.E. (2000). Peroxisome proliferator-activated receptor alpha-null mice lack resistance to acetaminophen hepatotoxicity following clofibrate exposure. Toxicol. Sci..

[9] Manautou J.E., Hoivik D.J., Tveit A., Hart S.G., Khairallah E.A., Cohen S.D. (1994). Clofibrate pretreatment diminishes acetaminophen's selective covalent binding and hepatotoxicity. Toxicol. Appl. Pharmacol..

[10] Shankar K., Vaidya V.S., Corton J.C., Bucci T.J., Liu J., Waalkes M.P., Mehendale H.M. (2003). Activation of PPAR-alpha in streptozotocin-induced diabetes is essential for resistance against acetaminophen toxicity. Faseb. J..

[11] Manautou J.E., Tveit A., Hoivik D.J., Khairallah E.A., Cohen S.D. (1996). Protection by clofibrate against acetaminophen hepatotoxicity in male CD-1 mice is associated with an early increase in biliary concentration of acetaminophen-glutathione adducts. Toxicol. Appl. Pharmacol..

[12] Manautou J.E., Emeigh Hart S.G., Khairallah E.A., Cohen S.D. (1996). Protection against acetaminophen hepatotoxicity by a single dose of clofibrate: Effects on selective protein arylation and glutathione depletion. Fundam. Appl. Toxicol..

[13] Donthamsetty S., Bhave V.S., Mitra M.S., Latendresse J.R., Mehendale H.M. (2008). Nonalcoholic steatohepatitic (NASH) mice are protected from higher hepatotoxicity of acetaminophen upon induction of PPARalpha with clofibrate. Toxicol. Appl. Pharmacol..

[14] Kleemann R., Verschuren L., de Rooij B.J., Lindeman J., de Maat M.M., Szalai A.J., Princen H.M., Kooistra T. (2004). Evidence for anti-inflammatory activity of statins and PPARalpha activators in human C-reactive protein transgenic mice *in vivo* and in cultured human hepatocytes *in vitro*. Blood.

[15] Chen C., Krausz K.W., Idle J.R., Gonzalez F.J. (2008). Identification of novel toxicity-associated metabolites by metabolomics and mass isotopomer analysis of acetaminophen metabolism in wild-type and Cyp2e1-null mice. J. Biol. Chem..

[16] Chen C., Krausz K.W., Shah Y.M., Idle J.R., Gonzalez F.J. (2009). Serum metabolomics reveals irreversible inhibition of fatty acid beta-oxidation through the suppression of PPARalpha activation as a contributing mechanism of acetaminophen-induced hepatotoxicity. Chem. Res. Toxicol..

[17] Coen M., Lenz E.M., Nicholson J.K., Wilson I.D., Pognan F., Lindon J.C. (2003). An integrated metabonomic investigation of acetaminophen toxicity in the mouse using NMR spectroscopy. Chem. Res. Toxicol..

[18] Arafa H.M. (2009). Carnitine deficiency: A possible risk factor in paracetamol hepatotoxicity. Arch. Toxicol..

[19] Yapar K., Kart A., Karapehlivan M., Atakisi O., Tunca R., Erginsoy S., Citil M. (2007). Hepatoprotective effect of L-carnitine against acute acetaminophen toxicity in mice. Exp. Toxicol. Pathol..

[20] Davern T.J., James L.P., Hinson J.A., Polson J., Larson A.M., Fontana R.J., Lalani E., Munoz S., Shakil A.O., Lee W.M. (2006). Measurement of serum acetaminophen-protein adducts in patients with acute liver failure. Gastroenterology.

[21] James L.P., Letzig L., Simpson P.M., Capparelli E., Roberts D.W., Hinson J.A., Davern T.J., Lee W.M. (2009). Pharmacokinetics of acetaminophen-protein adducts in adults with acetaminophen overdose and acute liver failure. Drug Metab. Dispos..

[22] Muldrew K.L., James L.P., Coop L., McCullough S.S., Hendrickson H.P., Hinson J.A., Mayeux P.R. (2002). Determination of acetaminophen-protein adducts in mouse liver and serum and human serum after hepatotoxic doses of acetaminophen using high- performance liquid chromatography with electrochemical detection. Drug Metab. Dispos..

[23] Donahower B., McCullough S.S., Kurten R.C., Lamps L.W., Simpson P.M., Hinson J.A., James L.P. (2006). Vascular Endothelial Growth Factor and Hepatocyte Regeneration in Acetaminophen Toxicity. Am. J. Physiol. Gastrointest Liver Physiol..

[24] James L.P., McCullough S.S., Lamps L.W., Hinson J.A. (2003). Effect of *N-*acetylcysteine on acetaminophen toxicity in mice: Relationship to reactive nitrogen and cytokine formation. Toxicol. Sci..

[25] Donahower B.C., McCullough S.S., Hennings L., Simpson P.M., Stowe C.D., Saad A.G., Kurten R.C., Hinson J.A., James L.P. (2010). Human recombinant vascular endothelial growth factor reduces necrosis and enhances hepatocyte regeneration in a mouse model of acetaminophen toxicity. J. Pharmacol. Exp. Ther..

[26] Buttar H.S., Nera E.A., Downie R.H. (1976). Serum enzyme activities and hepatic triglyceride levels in acute and subacute acetaminophen-treated rats. Toxicology.

[27] Nakatani T., Ozawa K., Asano M., Ukikusa M., Kamiyama Y., Tobe T. (1981). Differences in predominant energy substrate in relation to the resected hepatic mass in the phase immediately after hepatectomy. J. Lab. Clin. Med..

[28] Severino V., Locker J., Ledda-Columbano G.M., Columbano A., Parente A., Chambery A. (2011). Proteomic characterization of early changes induced by triiodothyronine in rat liver. J. Proteome Res..

[29] Mitchell J.R., Thorgeirsson S.S., Potter W.Z., Jollow D.J., Keiser H. (1974). Acetaminophen-induced hepatic injury: protective role of glutathione in man and rationale for therapy. Clin. Pharmacol. Ther..

[30] Chaudhuri S., McCullough S.S., Hennings L., Letzig L., Simpson P.M., Hinson J.A., James L.P. (2010). Acetaminophen hepatotoxicity and HIF-1alpha induction in mice occur without hypoxia. Toxicol. Appl. Pharmacol..

[31] Baumgardner J.N., Shankar K., Hennings L., Albano E., Badger T.M., Ronis M.J. (2008). *N-*acetylcysteine attenuates progression of liver pathology in a rat model of nonalcoholic steatohepatitis. J. Nutr..

[32] Varmuza K., Filzmoser P. (2009). Introduction to Multivariate Statistical Analysis in Chemometrics.

